# Supplementary figures and images for: Quantifying bacterial evolution in the wild: A birthday problem for Campylobacter lineages
Source: PLoS Genet. 2021 Sep 28;17(9):e1009829. doi: 10.1371/journal.pgen.1009829 (PMC8500405; doi:10.1371/journal.pgen.1009829)

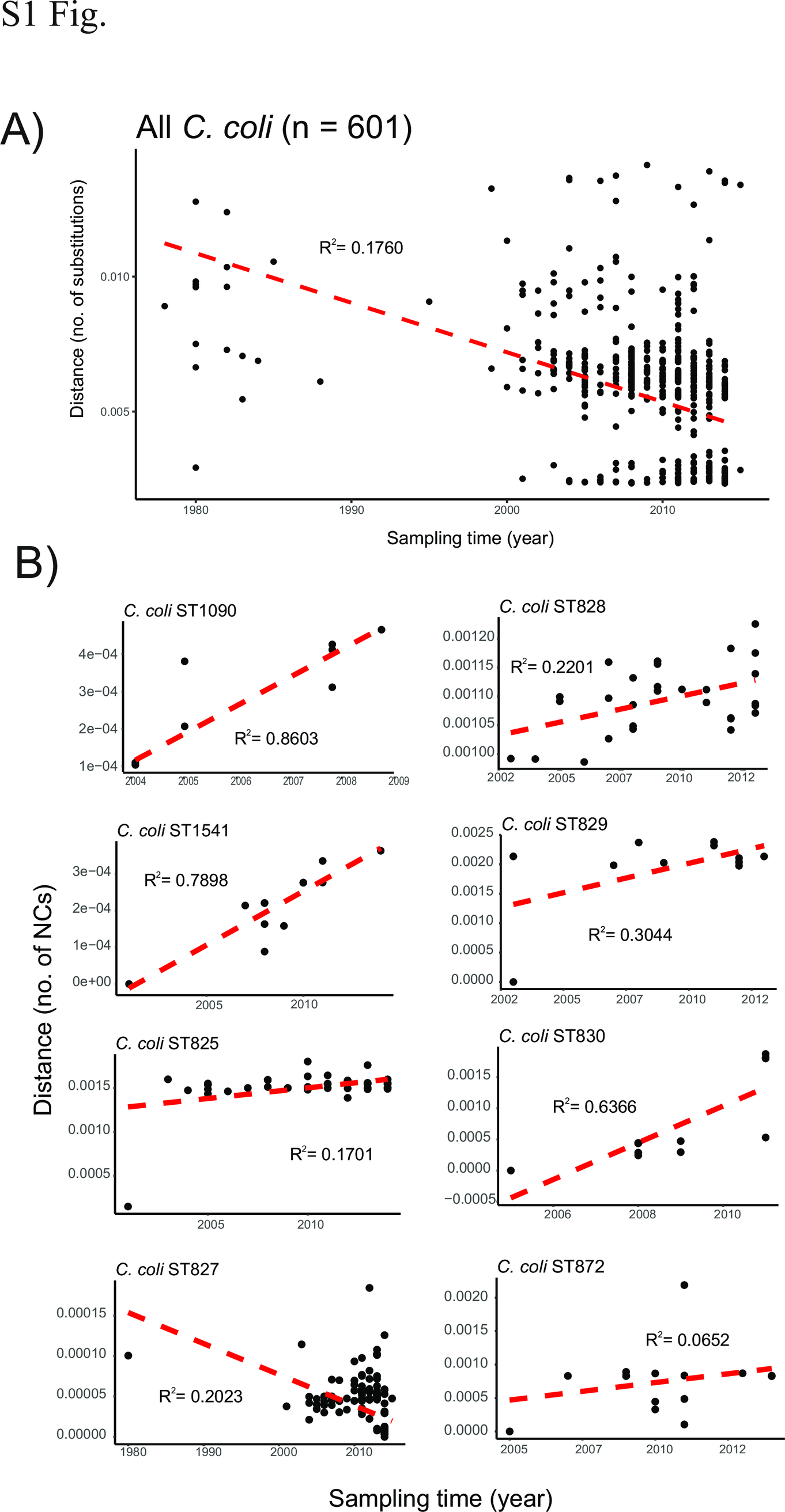

Supplement: S1 Fig — Root-to-tip genetic distance (y axis) is correlated with sampling time (x axis) for phylogenies of (A) 601 C. coli and (B) 8 sub-lineages of the ST-828 clonal complex. Only 3 out of 8 sub-lineages had strong temporal signal (R2 > 0.5). (TIF) [file pgen.1009829.s009.tif]

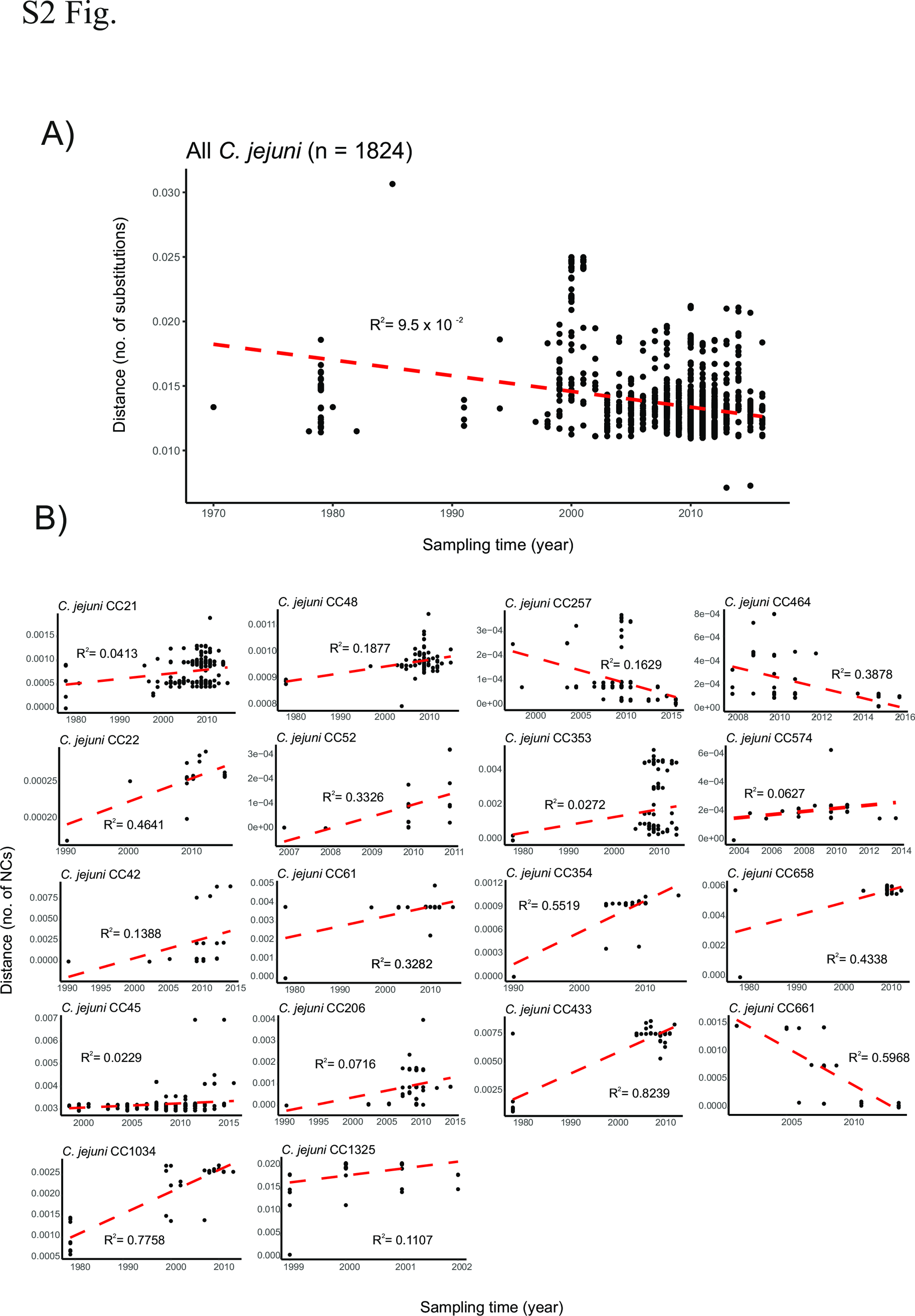

Supplement: S2 Fig — Root-to-tip genetic distance (y axis) is correlated with sampling time (x axis) for phylogenies of (A) 1824 C. jejuni and (B) 18 sub-lineages representing C. jejuni clonal complexes. Only 5 out of 18 sub-lineages had strong temporal signal (R2 > 0.5). (TIF) [file pgen.1009829.s010.tif]

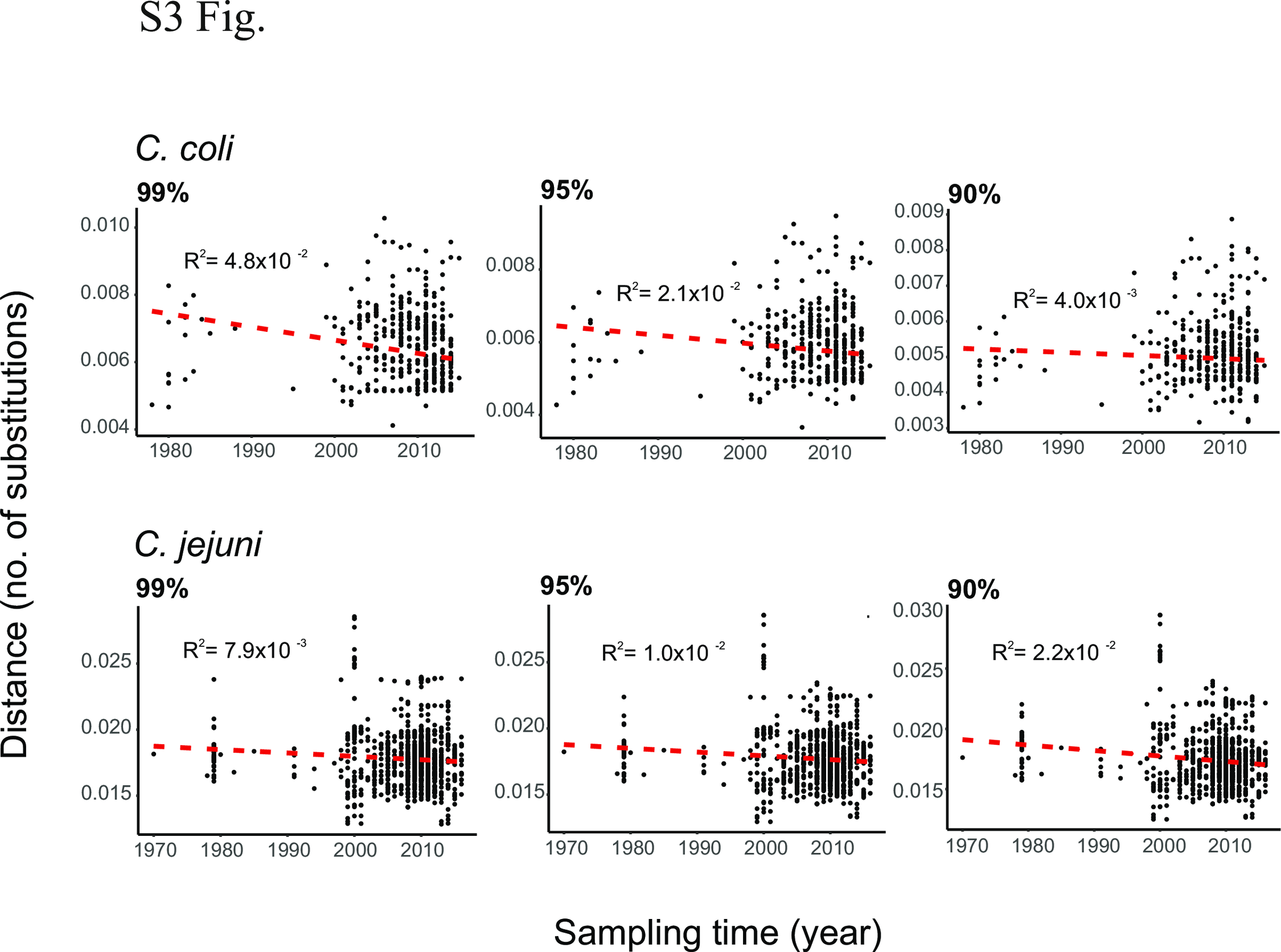

Supplement: S3 Fig — Core gene alignments were constructed from 601 (C. coli) and 1824 (C. jejuni) isolates. The most and least 1, 5 and 10% of variable loci (alleles/loci) were removed and phylogenies were analysed with Tempest. Root-to-tip genetic distance (y axis) is correlated with sampling time (x axis) to reveal poor temporal signal (R2 < 0.5) in all six scenarios. (TIF) [file pgen.1009829.s011.tif]

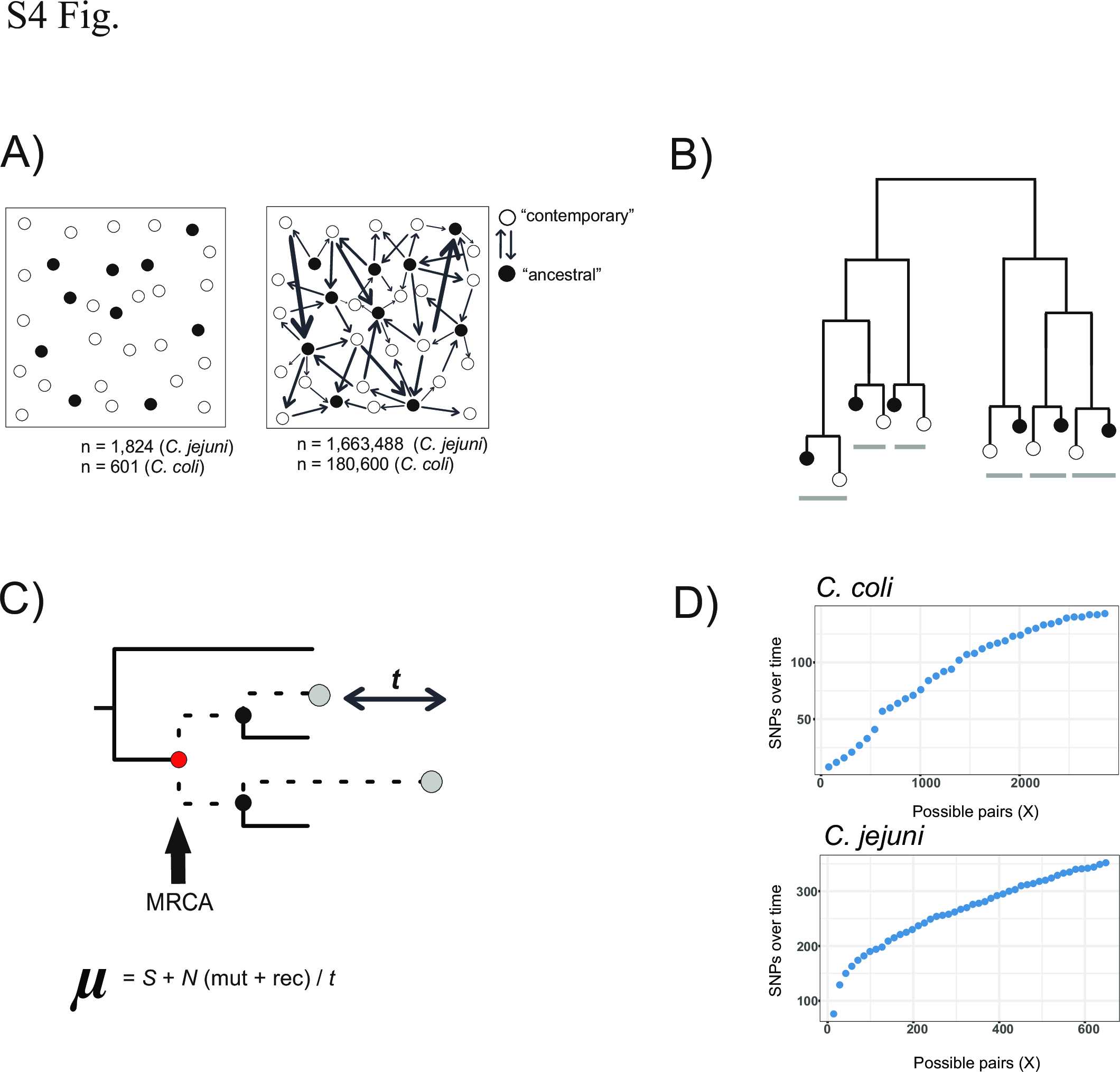

Supplement: S4 Fig — (A) The total number of C. jejuni and C. coli isolates in the population and all potential pairwise comparisons between putative ancestral (black) and contemporary (white) strains to give the total number of potential isolate pairs, Y. (B) Isolate pair selection based on divergent sampling date (>8 years) and a nucleotide identity threshold <5000 SNPs. (C) Total rate of nucleotide change (μ) calculated for all chosen pairs. The rate of accumulation of all synonymous (S), nonsynonymous (N) NCs, within (rec) and outside (mut) of recombined regions, was estimated since the most recent common ancestor (MRCA, red circle). The difference in NCs between each pair was divided by the difference in isolation years to give μ. (D) The total NC rate was used to estimate the number of NCs that were to accumulate over a time period and the number of possible isolate pairs at given time intervals (t1, t2,t3….tn) for each species. (TIF) [file pgen.1009829.s012.tif]

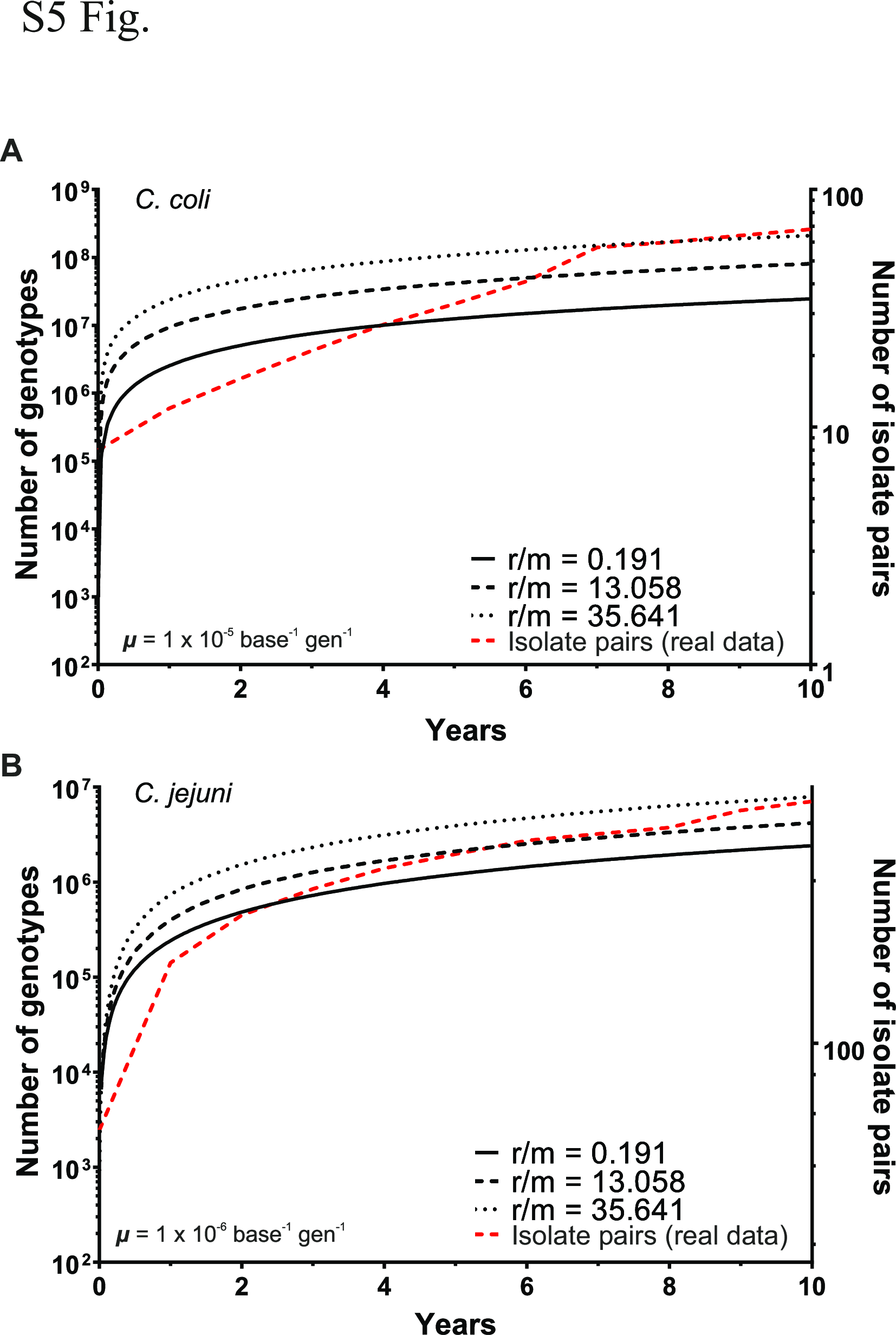

Supplement: S5 Fig — Plots show the increase in the number of genotypes (y axis) over 60,000 generations (x axis) representing ten years of Campylobacter doubling time for C. coli (A) and C. jejuni (B). Forward simulations were run with mutation rates reflecting estimates from this study and three different r/m values (high (35.641) (dotted line), medium (13.058) (dashed line), low (0.191) (block line)) to monitor the effects of recombination on genotype frequency from one generation to the next. Simulated data was compared to the number of possible isolate pairs with fewer SNPs than predicted based on the total NC rate (X) at a given time for C. coli and C. jejuni (red dashed line). (TIF) [file pgen.1009829.s013.tif]
